# Assessing children’s empathy through a Spanish adaptation of the Basic Empathy Scale: parent’s and child’s report forms

**DOI:** 10.3389/fpsyg.2014.01438

**Published:** 2014-12-15

**Authors:** Noelia Sánchez-Pérez, Luis J. Fuentes, Darrick Jolliffe, Carmen González-Salinas

**Affiliations:** ^1^Departamento de Psicología Evolutiva y de la Educación, Facultad de Psicología, Universidad de Murcia, MurciaSpain; ^2^Departamento de Psicología Básica y Metodología, Facultad de Psicología, Universidad de Murcia, MurciaSpain; ^3^Department of Law and Criminology, School of Law, University of Greenwich, LondonUK

**Keywords:** empathy, children, Basic Empathy Scale, self-report, parent-report

## Abstract

The aim of the current research was to study cognitive and affective empathy in children aged 6–12 years old, and their associations with children’s family environment and social adjustment. For this purpose, we developed the Spanish version of the Basic Empathy Scale (BES), self- and parent-report forms. Factorial analyses confirmed a two-component model of empathy in both self- and parent-report forms. Concordance between parent–child measures of empathy was low for cognitive and affective factors. Analyses of variance on the cognitive and affective components brought a significant effect of age for self-reported cognitive empathy, with older children scoring higher than younger ones. Gender brought out a significant principal effect for self-reported affective empathy, with girls scoring higher than boys. No other main effects were found for age and gender for the rest of the factors analyzed. Children’s empathy was associated with socioeconomic status and other family socialization processes, as well as children’ social behaviors. Overall the new measures provided a coherent view of empathy in middle childhood and early adolescence when measured through self and parent reports, and illustrate the similarity of the validity of the BES in a European-Spanish culture.

## INTRODUCTION

Empathy is an important interpersonal ability that contributes to the development of a variety of socio-emotional processes throughout childhood and into adolescence, such as prosocial and assertive behavior, social understanding, morality, and externalizing problems (Eisenberg and Miller, 1987; Miller and Eisenberg, 1988; Hoffman, 2000; Zhou et al., 2002; Findlay et al., 2006; Garaigordobil, 2009). The present study addresses children’s empathy, investigating its connections with family environment and social behaviors.

### CONCEPT AND STRUCTURE OF EMPATHY

In addressing the nature and structure of empathy, there is relative consensus that empathy is best understood as constituted of two dimensions (Baron-Cohen and Wheelwright, 2004; Lawrence et al., 2004; Jolliffe and Farrington, 2006; Shamay-Tsoory, 2011). One of them, *cognitive empathy*, is defined as the capacity to understand other’s feelings (Kohler, 1929; Hogan, 1969), and is a cognitive process through which a person constructs the mental state of another (Hogan, 1969; Blair, 2005). The other component, *affective empathy*, is the tendency to experience an emotionally concordant response to the affective state of another (Feshbach, 1975; Baron-Cohen and Wheelwright, 2004). These two empathy components have been integrated in the framework developed by (Eisenberg and Eggum, 2009; Eisenberg et al., 2010). Adopting a developmental perspective, they define empathy as “an affective response that stems from the apprehension or comprehension of another’s emotional state or condition, and is similar to what the other person is feeling or would be expected to feel” (Eisenberg, 2000, p. 671). Even though the emotional response is a central component of this conceptualization of empathy, the understanding of another’s emotional state is necessary for the development of empathy.

Clinical and cognitive neuroscience research is also generally supportive of this two-component model of empathy. From a clinical perspective, deficits in cognitive or affective empathy have been differentially associated with specific developmental and personality disorders. For instance, Asperger’s syndrome has been specifically associated with impairments in cognitive empathy (Rogers et al., 2007; Dziobek et al., 2008), while narcissistic personality disorder (Ritter et al., 2011) and psychopathic tendencies (Jones et al., 2010) have been linked to deficits in affective ability. Neuroimaging and lesion studies also point to different neural networks for each empathy component (Cox et al., 2012; see Shamay-Tsoory, 2011, for a review).

### THE MEASUREMENT OF EMPATHY IN CHILDHOOD AND ADOLESCENCE

Different methods have been used to measure empathy, such as direct and structured observation of behaviors and reactions in laboratory (Lennon et al., 1986; de Wied et al., 2005; Light et al., 2009), and neuroimaging techniques involving functional magnetic resonance imaging and event-related brain potential (Decety et al., 2008; Fan and Han, 2008; Han et al., 2008; Masten et al., 2011). However, self-reports constitute the most extensive strategy used for the study of empathy (Gerdes et al., 2010), in part because it involves less economical and technical investment while providing extensive information. As a disadvantage however, questionnaires can be affected by subjective biases (e.g., Eisenberg et al., 1991; Choi and Pak, 2005).

Among the questionnaires available to measure empathy in childhood and adolescence, the Basic Empathy Scale (BES; Jolliffe and Farrington, 2006) has a number of benefits. First, it was specifically developed based on the definition of empathy provided by Cohen and Strayer (1996), “the understanding and sharing in another’s emotional state or context” (p. 523), therefore allowing for the measurement of the two key components of empathy. Second, these two components have clear distinct operational definitions, avoiding the overlapping with other close concepts; in the case of *cognitive empathy* scale, items highlight the comprehension of another’s emotion and this approach allows differentiating cognitive empathy from perspective taking ability. In *affective empathy* scale, items emphasize the emotional congruence, with a distinction from empathy-related responding, such as sympathy. In third place, empathic responses are measured in the context of several primary emotional reactions, including both positive as well as negative emotions. In fourth place, and concerning items formulation, they have an easy wording, enabling people of a wide range of educational backgrounds to understand and complete the questionnaire. Lastly, and following Kline (1993), items were also generated avoiding emotive words that could induce empathic responses and therefore decreasing self-presentation bias and social desirability responses.

The development and validation of the BES was carried out originally on a sample of 720 English adolescents, and factor analyses supported the two-componential model, with separated cognitive and affective factors *versus* a one-dimensional model. Further studies developed in French, Italian, and Chinese languages have given support for the factorial model previously found, and indicate that BES preserves a good psychometric functioning in different cultures (Albiero et al., 2009; D’Ambrosio et al., 2009; Geng et al., 2012). The development of equivalent instruments for measuring the same constructs in a wide array of cultures would allow investigating the generality versus specificity of empathy development and its connections to social adjustment across cultures. The present work aims to contribute to this effort by developing an adaptation of the BES self-report into European-Spanish language and studying its psychometric properties in a sample of children aged 8–12 years.

By using self-report questionnaires we should be aware however that some people could present difficulties accessing or expressing how they or another person feel. This could be especially true for younger children, whose still developing cognitive and verbal abilities can make the task of reporting on internal states difficult (Dadds et al., 2008). In order to gain a more accurate measurement, a multi-informant or triangulated approach has been recommended (Dadds et al., 2008; Gerdes et al., 2010; Nelson and Harwood, 2011). In this respect, parents could constitute a helpful supplementary source of information about their children’s empathic behavior.

Although a multi-informant approach is clearly a desirable approach for the measurement of socio-emotional processes in childhood and adolescence, this strategy has been scarcely used in the study of children’s empathy. Cliffordson (2001) measured the empathy of children using the Interpersonal Reactivity Index (IRI; Davis, 1983) and also had parents report on their perceptions of their children’s empathy using the same measure. Although the four-factor structure of empathy of the IRI appeared in both parent and children samples (constituting of perspective taking, empathic concern, personal distress, and fantasy), the concordance between parent–child judgments, as measured *via* Pearson correlations, was low for most of the factors studied. Additionally, parents’ and children’s mean scores comparisons brought out non-significant differences for fantasy and personal distress scales, whereas empathic concern and perspective taking scales mean scores were higher in parents’ reports. Cliffordson (2001) interpreted this low concordance as accurate rather than a measurement error, and suggested that there were many difficulties involved in the measurement of internal states. Alternatively, discrepancies between judgments have been attributed to the contexts or situations in which different informants observe the child’s behavior (Achenbach et al., 1987).

In the light of the literature reviewed, it is apparent that more research involving multiple informants is necessary. Therefore, in addition to self-reports of empathy, this work aimed to evaluate parental perception of children’s empathy by developing and validating a BES parent-report form that covers a wide age range, including children as young as 6 years old and covering ages until 12 years.

### AGE AND GENDER DIFFERENCES IN CHILDREN’S EMPATHY

In exploring empathy from childhood and into adolescence, changes in empathy are expected. Early in middle childhood, children are already able to perform many of the cognitive processes necessary for the development of empathic skills, such as awareness of others, self-other differentiation and perspective taking ability (Ungerer et al., 1990; Decety, 2010). Further cognitive development fostered by the maturation of the prefrontal cortex (Diamond, 2002; Tsujimoto, 2008) and a higher level of social cognition (Saxe, 2006), would produce qualitative changes in empathy experience throughout time, achieving its highest developmental stage during late adolescence (Hoffman, 1987). Since the affective and cognitive components involve interacting yet partially non-overlapping neural circuits that undergo changes at a different rate with age, most scholars agree that they have different developmental trajectories (e.g., Hodges and Klein, 2001; Decety and Jackson, 2004; Eisenberg and Eggum, 2009). More specifically, the affective component – which involves limbic areas among other structures – would asymptote early in childhood, while the cognitive component – more dependent on frontal lobe – would show changes from childhood and into adolescence (Decety, 2010). Empirical research addressing age differences has found, nonetheless, inconsistent findings that have varied with the age-period covered and the specific measurement instrument used. For instance, Dadds et al. (2008) found that children’s cognitive empathy (reported by parents) increased throughout childhood and adolescence (from 4 to 16 years old), while no age differences were found for affective empathy. In contrast, using self-reports in a sample of adolescents aged 9 to 18 years old, Geng et al. (2012) found that both cognitive and affective empathy increased with age, although the effect sizes were small. Given these inconsistencies and the relatively small amount of research on the variation in the two components of empathy throughout childhood and into adolescence, age-related changes are considered in this study.

Along with age, gender has been identified as a relevant factor explaining individual differences in empathy skills. Females have consistently scored higher on measures of empathy, particularly on affective empathy (Baron-Cohen and Wheelwright, 2004; Jolliffe and Farrington, 2006; D’Ambrosio et al., 2009; Geng et al., 2012). However, the magnitude of the female–male differences on measures of empathy appears to vary depending on the age period studied, with greater boy–girl differences for older ages (Mestre et al., 2009), and as a function of the measurement method (Eisenberg and Lennon, 1983). Because of the importance of the gender condition in understanding the development of empathy, the potential impact of this variable is taken into account in this study, and girls are expected to score higher than boys, especially in affective empathy. Nevertheless, given the early ages considered in this work, gender differences could be small.

### CHILDREN’S EMPATHY: FAMILY INFLUENCES AND SOCIAL BEHAVIORS

In understanding individual differences in empathy during childhood and adolescence, low socioeconomic status (SES) and other potential family influences – especially those related to affect – have also been found relevant. More specifically, low SES has been associated with lower levels of empathy (Jolliffe and Farrington, 2006). Other family influences include maternal support (Soenens et al., 2007), high-quality parenting (Laible et al., 2004), warm parenting (Barnett, 1987), and siblings’ warmth (Lam et al., 2012). In connection with that body of results, the present study also addresses the relationship of family environment with children’s empathy.

In turn, individual differences in empathy can explain in part the quality of social adjustment in childhood and could have an impact on behavior. Previous research has shown that children with higher levels of empathy show higher scores on social competence, prosocial behavior, and are more accepted among peers (Eisenberg and Miller, 1987; Davis, 1996; Albiero et al., 2009; Eisenberg et al., 2010; Taylor et al., 2013). Yet, the relation between empathy and aggression and antisocial behavior is not clear. As theorized, some studies have indicated that empathy is negatively related to aggression and disruptive behavior (Miller and Eisenberg, 1988; de Wied et al., 2005), while others have noted that these relations are weak (Vachon et al., 2013) or inconsistent (Lovett and Sheffield, 2007). These contradictory findings have been attributed, among other causes, to differences in the studies with respect to the kind of measurement tool used, the contrast between overt *versus* relational aggression, and the developmental period studied (Miller and Eisenberg, 1988; Lovett and Sheffield, 2007; Geng et al., 2012; Batanova and Loukas, 2013). As more research is still needed, we have included measures of social behaviors (social skills and aggression) in which to compare self and parents’ perceptions of children’s empathy.

In summary, this work aims to study both cognitive and affective empathy in a sample of Spaniards aged 6 to 12 years. For that purpose, we adapted the BES into European-Spanish language, with two forms: self- and parent- report. The psychometric properties and factorial structure of both forms in this sample are examined. In addition, the potential influence of age and gender are also taken into account. Lastly, the associations of individual differences in empathy with children’ SES and other family socialization processes, as well as social behaviors, are also explored.

## MATERIALS AND METHODS

### PARTICIPANTS

#### Parents filling out the BES-PR

This sample was drawn from a broader study about the cognitive, social and emotional factors that influence children’s adjustment to school (MATES Project, PSI2011-23340). It was initially composed of 457 families but, for validation purposes, 93 participants (20.4%) were excluded for various reasons, including low Spanish language ability (16 participants), incomplete BES (five participants), child diagnosed with learning disability or clinical problems (47) and children under age 6 (four participants). Also, for those families where two or more siblings were involved in the initial sample, we randomly removed a sibling (21), retaining only one child per family. Therefore, a total of 364 valid cases (182 boys and 182 girls) aged 6 to 12 years (*M* = 9.14, SD = 1.75) were finally included in the study. All children were enrolled in Primary School Education in one of the nine schools in the Region of Murcia (Spain) that participated in the study.

The mothers were aged 21–57 years old (*M* = 39.67, SD = 5.29). Slightly less than half (45.7%) were educated up to elementary school level, 25.5% to high school, and 28.8% to university level. Fathers’ age ranged from 26 to 62 (*M* = 42.34, SD = 5.43). The level of father’s education was similar to that of mothers (46.3% were educated to elementary school level, 28.6% to high school and 25.1% to university).

The questionnaires about children were completed mostly by mothers (68.5%), or jointly by mothers and fathers (21.8%). Only a relatively small number were completed by fathers alone (9.1%) or by another legal guardian (0.6%). Most parents came from European backgrounds (91.1%), with the remainder coming from Latin American (4.7%), African (3.3%), or Gypsy (0.9%) origins. That demographic distribution broadly reflects the ethnic variability of the local geographic area. With respect to family structure, children lived with both parents in most cases (88.6%), and the rest lived with their mother or father solely (11.4%).

Families were also asked about their monthly income levels, ranging from “less than 750€” to “more than 3000€.” Approximately, 16.2% of the parents did not complete this question. Among the parents who responded, 11.8% reported their incomes to be less than 750€ per month, 19.3% reported between 751 and 1200€, 17.1% reported between 1201 and 1600€, 10.8% reported between 1601 and 2000€, 27.9% reported between 2001 and 3000€ and 13.1% of the parents reported more than 3000€.

#### Children filling out the Basic Empathy Scale-self report (BES-SR)

The Spanish BES was administered to a total of 290 children (145 boys, 145 girls) aged 8–12 years (*M* = 9.96, SD = 1.17). Although the parents’ sample included a group of 6–7 year-old children, the self-report scale was administered to children older than 8 years old. This is following Dadds’ recommendation (Dadds et al., 2008).

### MEASURES

#### The Basic Empathy Scale-self report

Children aged 8 years or older completed a version of BES translated into Spanish and back translated. The BES comprises a total of 20 items that measure both cognitive and affective empathy. Children were asked to report the extent to which they agreed or disagreed with each statement using a five-point Likert scale that ranged from 1 (“Strongly disagree”) to 5 (“Strongly agree”). Scores on cognitive and affective empathy scales were calculated by dividing the total score by the number of responded items included in each scale. This scoring strategy is suitable for managing answered items in the questionnaire (Putnam and Rothbart, 2006).

#### The Basic Empathy Scale-parent report (BES-PR)

Basic Empathy Scale-parent report (BES-PR) items were taken from the original BES and reworded in third person, retaining the original content. For example, the Basic Empathy Scale-self report (BES-SR) item “I get caught up in other people’s feelings easily,” was reworded as: “S/he gets caught up in other people’s feelings easily.” Parents were asked about the extent to which they agreed or disagreed with each statement using a Likert-type scale ranging from 1 (“Strongly disagree”) to 5 (“Strongly agree”). An additional option of “Not Applicable” was provided so that parents could inform of not being able to observe their children in the specified situation. Scores of the affective and cognitive scales were calculated following the same strategy as the BES-SR.

#### Positive family climate

Positive family climate was measured using the *relationship* dimension of the Family Environment Scale (FES; Moos et al., 1984; Spanish version developed by Seisdedos et al., 1989). This 27-item device measures the degree of commitment and support family members provide for one another, the extent to which family members are encouraged to express their feelings, and the level of harmony in contrast to conflict between family members. Each sentence was scored as true (scored as 1) or false (scored as 0) by parents. The KR-20 Kuder–Richardson coefficient of this scale was 0.71.

#### Dissatisfaction with family relationships

Children completed the scales *dissatisfaction with siblings*^1^, which measures the degree of dissatisfaction in relation to siblings through jealousy, squabbling, differences and internal conflicts, and *dissatisfaction with family environment,* which measures the degree of dissatisfaction with general home climate and the relationship between parents. These scales were taken from the Test Autoevaluativo Multifactorial de Adaptación Infantil (TAMAI; Hernández-Guanir, 2009). These scales were combined and standardized to create an overall measure of dissatisfaction with family relationships. The KR-20 Kuder–Richardson coefficient was 0.69.

#### Weak parental management

Weak parental management was measured using six items from the “Social and Familiar Life Stressors Inventory” (González-Salinas and Sánchez-Perez, unpublished manuscript), completed by the children’s teacher. Teachers were asked to identify the frequency, ranging from 1 (never) to 4 (always), with which children showed signs of weak supervision/care from parents. Example items are “does not bring back the homework,” and “comes from home unclean.” Cronbach’s alpha for this measure was 0.75.

#### Socioeconomic status

Parents reported their monthly family income. This was recorded as an ordinal scale ranging from 1 (“less than 750€”) to 5 (“more than 3000€”). The mean score was 3.61 (SD = 1.64).

#### Social behaviors

Children’s social skills and aggressive behavior were reported by parents and teachers using *social skills* and *aggression* scales respectively taken from the Behavior Assessment System for Children (BASC; Reynolds and Kamphaus, 2004; Spanish version developed by González et al., 2004). This scale provides a measure of the frequency, ranging from 1 (never) to 4 (almost always), with which children are viewed to successfully interact with peers and adults in the contexts of home, school, and community (social skills), and the extent to which children show some tendencies to act in a hostile manner (verbally or physically) that threatens others (aggression). A composite score of social skills and aggression was formed respectively by standardizing and averaging the scores provided by parents and teachers. Cronbach’s alpha was 0.88 for social skills and 0.91 for aggression.

**Table 1** shows the means, standard deviations, number of cases, and who was the informant for the measures included in this study.

**Table 1:** Means and standard deviations for the variables under study.

	*M*	SD	*N*	Reported by
Cognitive empathy (BES-SR)	3.89	0.63	290	Children
Affective empathy (BES-SR)	3.12	0.63	290	Children
Cognitive empathy (BES-PR)	4.09	0.49	364	Parents
Affective empathy (BES-PR)	3.88	0.59	364	Parents
Positive family climate	20.23	3.33	124	Parents
Dissatisfaction with family relationships	0.03	0.89	113	Children
Weak parental management	1.22	0.31	101	Teachers
Socioeconomic status (parents’ sample)	3.63	1.64	305	Parents
Social skills	0.01	0.81	237	Parents and teachers
Aggression	-0.01	0.83	237	Parents and teachers

### PROCEDURE

We first contacted the authorities of nine Primary Schools in the Region of Murcia, and once approval was granted, letters describing the research project and consent forms were delivered to families. Parents who consented received the Spanish version of BES-PR and a questionnaire asking about basic socio-demographic information, which they were then asked to return to the school. A member of the research team was available at the school to answer any questions or concerns raised by the parents. Once the parents returned the questionnaires, they were given the BASC and the FES to be completed again at home, following the procedure specified above.

Children aged 8 or older completed the BES-SR and TAMAI in a small room of their school assigned by the head-teacher. In order to address potential issues of literacy amongst children, the items were read aloud to groups of approximately 10 children. Questionnaire administration took about 30 min.

Teachers completed the BASC and the parental management questionnaire following instructions given by a person of our staff. One of the teachers refused to give information about poor parental management, so that 12 questionnaires kept unfulfilled.

## RESULTS

### TESTING THE FACTORIAL STRUCTURE OF BES SELF AND PARENT REPORTS

#### Items response frequency

As the BES was originally developed for adolescents and adults, the proportion of NA responses for every item of the parent-report form was calculated to test the adequacy of every item in each age group. The sample was divided into four age groups of similar number size on whom the children were reporting, 6–7 year-olds (N = 80), 8–9 (N = 109), 10 (N = 81), and 11–12 (N = 94). The mean percentage of NA was low in general (M = 2.95%, ranging from 1.10 to 8.79%) and equally distributed across items and age groups. Only the items 4 and 6 were identified with a higher frequency of “Not Applicable” compared to others. In the case of sentence 4, this item showed a NA frequency of 15% of the cases for the younger age group. It is worth mentioning that this frequency lowered to 6.38% for the older group. In the case of item 6, the higher NA frequency of “Not Applicable” (12.5%) was located in the 10 years group and also casts doubts about its suitability for the ages involved in the present study.

#### Internal consistency

In the BES-SR, item-scale correlations ranged from *r* = 0.26 to *r* = 0.52 for the cognitive empathy scale and from *r* = 0.12 to *r* = 0.49 for the affective empathy scale (see **Table 2**). Two items of the affective empathy scale were excluded because they correlated under *r* = 0.20. These were items 4 and 7.

**Table 2:** Item-test correlations for BES self-report.

		Item-test correlation	
*N*∘	Item	Cognitive scale	Affective scale
1	My friend’s emotions don’t affect me much		0.20
2	After being with a friend who is sad about something, I usually feel sad		0.28
4	I get frightened when I watch characters in a good scary movie		0.12
5	I get caught up in other people’s feelings easily		0.49
7	I don’t become sad when I see other people crying		0.15
8	Other people’s feelings don’t bother me at all		0.27
11	I often become sad when watching sad things on TV or in films		0.31
13	Seeing a person who has been angered has no effect on my feelings		0.25
15	I tend to feel scared when I am with friends who are afraid		0.40
17	I often get swept up in my friend’s feelings		0.46
18	My friend’s unhappiness doesn’t make me feel anything		0.38
3	I can understand my friend’s happiness when s/he does well at something	0.26	
6	I find it hard to know when my friends are frightened	0.29	
9	When someone is feeling ‘down’ I can usually understand how s/he feels	0.36	
10	I can usually work out when my friends are scared	0.36	
12	I can often understand how people are feeling even before they tell me	0.44	
14	I can usually work out when people are cheerful	0.46	
16	I can usually realize quickly when a friend is angry	0.52	
19	I am not usually aware of my friend’s feelings	0.42	
20	I have trouble figuring out when my friends are happy	0.33	

The BES-PR item-scale correlations ranged from *r* = 0.18 to 0.56 for the cognitive empathy scale, and from *r* = 0.20 to 0.52 for affective empathy scale. An item of the cognitive empathy scale was excluded because it correlated under *r* = 0.20. This was the item 6 (see **Table 3**).

**Table 3:** Item-test correlations for BES parent report.

		Item-test correlation	
*N*∘	Item	Cognitive scale	Affective scale
1	Her/his parents’ or siblings’ emotions don’t affect her/him much		0.20
2	After being with a relative who is sad about something, s/he usually feels sad		0.40
4	S/he gets frightened when s/he watches characters in a good scary movie		0.32
5	S/he gets caught up in other people’s feelings easily		0.52
7	S/he doesn’t become sad when he/she sees other people crying		0.42
8	Other people’s feelings don’t bother her/him at all		0.47
11	S/he often becomes sad when watching sad things on TV or in films		0.50
13	Seeing a person who has been angered has no effect on her/his feelings		0.40
15	S/he tends to feel scared when s/he is with friends or relatives who are afraid		0.23
17	S/he often gets swept up in her/his siblings’ or friend’s feelings		0.39
18	Her/his relatives or friend’s unhappiness doesn’t make her/him feels anything		0.48
3	S/he can understand her/his friend’ or relative’s happiness when that person does well at something	0.38	
6	S/he finds it hard to know when other children are frightened	0.18	
9	When someone is feeling ‘down’ s/he can usually understand how that person feels	0.56	
10	S/he can usually work out when other children are scared	0.50	
12	S/he can often understand how people are feeling even before they tell her/him	0.46	
14	S/he can usually work out when people are cheerful	0.48	
16	S/he can usually realize quickly when either of her/his parents is angry	0.37	
19	S/he is not usually aware of her/his loved ones’ feelings	0.47	
20	S/he has trouble figuring out when other members of the family are happy	0.42	

For the BES-SR, Cronbach’s alpha coefficients were 0.70 for cognitive scale, and 0.66 for affective scale; Cronbach’s alpha coefficients of the BES-PR were 0.76 for cognitive and 0.74 for Affective scale.

#### Construct validity

To examine the goodness of fit of the two-factor model obtained in the original BES, confirmatory factor analysis (CFA) was performed with RStudio (R Core Team, 2012). The resulting models were obtained using diagonally weighted least squares (DWLS) method because the BES questionnaire consists of Likert-type scale items, and this estimator method provides more accurate parameter estimates when variables are ordinal (Mîndrilă, 2010). Since cognitive and affective empathy scales were correlated in both report forms, we specified the CFAs as oblique models, implying that factor loadings are regression coefficients (not correlations), which could reach values larger than 1 in magnitude (Jöreskog, 1999).

To obtain every latent factor, we selected the item with the highest item-test correlation in each scale as the first indicator, setting its factor loading to 1. For the self-report form, these items were number 16 and 5 for cognitive and affective empathy scales respectively; for parent-report, items 9 and 5 were selected for cognitive and affective empathy scales respectively.

Multiple indicators can be useful in evaluating goodness of fit. In the current study, five goodness-of-fit indices were used to assess the adequacy of each model fit: Chi-squared divided by the degrees of freedom (χ^2^/df), the Bentler comparative fit index (CFI), the Tucker-Lewis index (TLI), the root mean square error of approximation (RMSEA), and the standardized root mean squared residual (SRMR).

The 18-item BES-SR produced satisfactory fit indices: Chi squared divided by the degrees of freedom (χ^2^/df) was 1.52, CFI and TLI were 0.93 and 0.92 respectively, RMSEA was 0.04 and SRMR was 0.07. Therefore the CFA supports a two-factor solution for BES self-report in this sample.

Results of the CFA for the BES-PR revealed that the model with 19 items did not fit well. The fit for two alternative models was subsequently investigated, one with 18 items (deleting item 4, as for BES-SR), and another model with 17 items (removing item 7, also deleted in BES-SR). Given the fit indices of the three models (**Table 4**), a two-factor model with 17 items for the BES-PR was considered acceptable (Cronbach’s alpha for the modified affective empathy scale was 0.73)

**Table 4:** Goodness of fit indices for the three models of BES parent report.

BES parent report	χ^2^	df	CFI	TLI	RMSEA	SRMR
19 item model	359.15	151	0.86	0.84	0.07	0.11
18 item model	285.46	134	0.90	0.89	0.06	0.10
17 item model	223.99	118	0.93	0.92	0.06	0.10

As in the original work, CFA were computed separately by gender for both the BES-SR and BES-PR. For the BES-SR, goodness-of fit indices were: χ^2^/df = 1.08, boys/1.21, girls; CFI = 0.97, boys/0.94, girls; TLI = 0.97, boys/0.93, girls; RMSEA = 0.03, boys/0.04, girls; SRMR = 0.09, boys/0.09, girls. For the BES-PR, results were: χ^2^/df = 1.34, boys/1.19, girls; CFI = 0.95, boys/0.97, girls; TLI = 0.94, boys/0.97, girls; RMSEA = 0.05, boys/0.04, girls; SRMR = 0.11, boys/0.11, girls. These results provide support for the two-factor solution of the BES.

Associations between cognitive and affective empathy were significant and positive for both the BES-SR (*r* = 0.32, *p* < 0.001) and the BES-PR (*r* = 0.58, *p* < 0.001). These inter-correlations suggest that cognitive and affective empathy are related but separate dimensions.

**FIGURE 1:**
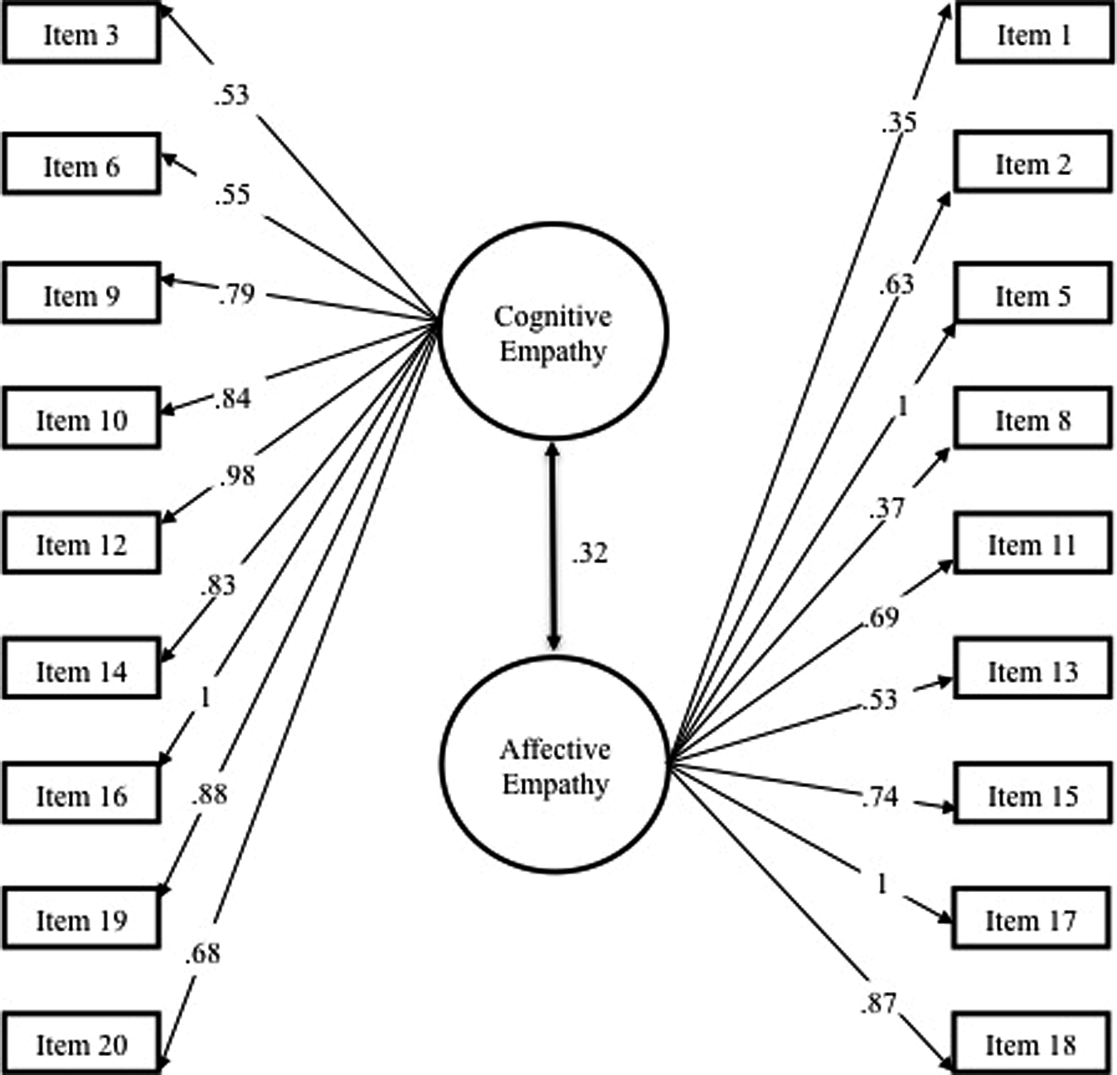
**Factor loadings for CFA and intercorrelations for BES self-report.** Beta coefficients larger than 1 have been set to 1 for interpretation purposes.

**FIGURE 2:**
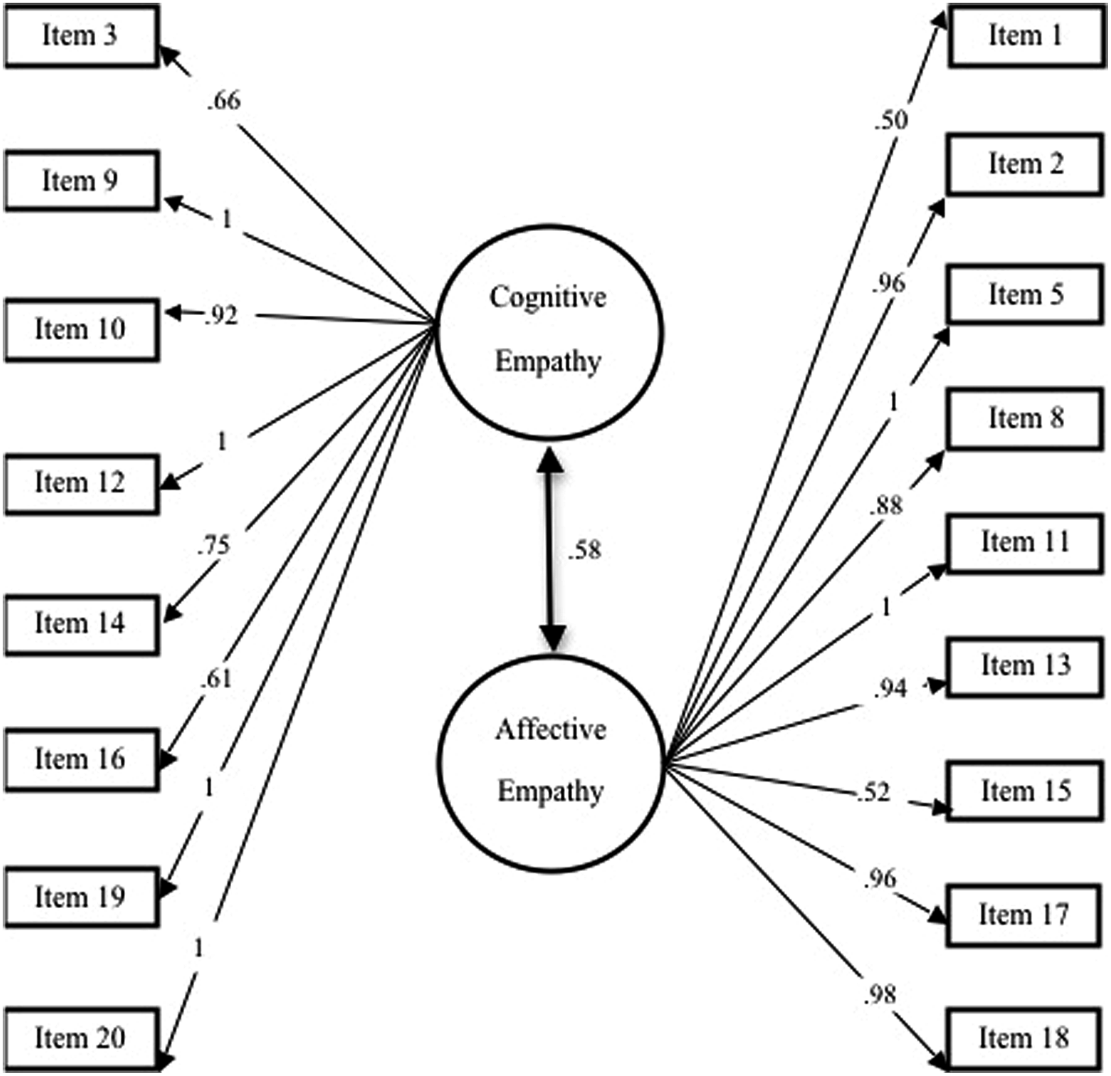
**Factor loadings for CFA and intercorrelations for BES parent report.** Beta coefficients larger than 1 have been set to 1 for interpretation purposes.

The two-factor models for the BES-SR and BES-PR are presented in **Figures 1** and **2**, respectively.

Spearman correlations were conducted to examine the degree of agreement between parents and children in empathy scores. Results showed a low association between cognitive (ρ = 0.10, *p* = 0.110) and affective empathy (ρ = 0.16, *p* = 0.006) self- and parent report. Mean scores provided by parents and children were also compared through paired *t*-tests. Results showed that parents scored higher than children in cognitive empathy (*t* = 3.79; *p* < 0.001) as well as affective empathy (*t* = 15.24; *p* < 0.001). In a further study of the influence of age and gender in the degree of parent–child agreement, a new variable was created calculating the absolute difference between parents’ and children’s scores in every empathy scale. A mixed 2 × 3 analysis of variance (ANOVA) including gender and age as between factors was run. This technique did not show any significant effect.

### TESTING THE CONSTRUCT VALIDITY OF THE BES-SR AND BES-PR

#### Gender and age differences

Analysis of variances were run on children’s reports, taking gender as the between subject variable and age as covariate. For cognitive empathy, only a significant main effect of age was found [*F*(1,287) = 5.58, *p* = 0.019, $\eta_{p}^{2}$ = 0.019]. Further analysis of this effect brought out a positive Pearson correlation of *r* = 0.14, *p* = 0.019. This involves that children scored higher in cognitive empathy as they grew older. For affective empathy, only a significant main effect of gender was found [*F*(1,287) = 9.52, *p* = 0.002, $\eta_{p}^{2}$ = 0.032], with girls scoring significantly higher than boys.

A similar procedure was used to investigate the influence of both gender and age on empathy scales through the BES-PR. No significant effects were found for either gender or age in cognitive and affective empathy scales.

Means and standard deviations for the BES-SR and BES-PR comparison groups are provided in **Table 5**.

**Table 5:** Means and standard deviations for the BES self- and parent-report split by gender.

Informant	Scale	Boys		Girls		Total	
		*M*	SD	*M*	SD	*M*	SD
Child	Cognitive empathy	3.89	0.60	3.89	0.65	3.89	0.63
	Affective empathy	2.98	0.72	3.23	0.64	3.10	0.69
Parent	Cognitive empathy	4.15	0.52	4.15	0.50	4.15	0.51
	Affective empathy	3.85	0.60	3.91	0.58	3.88	0.59

#### Empathy-family variables correlations

Correlations between the affective and cognitive scales of the BES (both SR and PR) and measures of family climate, family dissatisfaction and parental management are presented in **Table 6**. Given that these variables did not meet the assumptions of normality or were ordinal variables, Spearman correlations were employed.

**Table 6:** Zero-order correlations for empathy and family variables.

Measure	Positive family climate (*N*)	Dissatisfaction with family relationship (*N*)	Weak parental management (*N*)	SES (*N*)
Cognitive empathy (BES-SR)	0.01 (90)	-0.18^†^ (113)	-0.08 (101)	0.19** (242)
Affective empathy (BES-SR)	-0.05 (90)	-0.25* (113)	-0.04 (101)	0.12^†^ (242)
Cognitive empathy (BES-PR)	0.20* (124)	-0.17^†^ (113)	-0.14 (101)	0.16** (305)
Affective empathy (BES-PR)	0.14 (124)	-0.01 (113)	-0.28** (101)	0.19** (305)

^†^*p* < 0.10; *p < 0.05; **p < 0.01.

The resulting correlation coefficients suggested that the BES-SR was not significantly related to family climate or parental management. However, children who were more satisfied with their family scored significantly higher on affective empathy, and children who came from higher SES families had significantly higher scores on cognitive empathy. There was some indication that higher self-reported cognitive empathy was associated with greater satisfaction with one’s family and higher affective empathy was associated with higher family SES although the correlations were marginally significant.

Findings for the BES-PR in relation to the family variables were similar to those of the BES-SR. Both cognitive and affective empathy were associated with higher SES, and there was some indication that those perceived to have higher cognitive empathy were more satisfied with their family. Additionally, parental reported affective empathy was negatively related to weak parental management, and cognitive empathy was positively related to family climate.

#### Empathy-social behaviors correlations

Correlations between cognitive and affective scales with social skills and aggression (see **Table 7**) suggested that social skills were associated positively with cognitive and affective empathy for both self and parental reports (although this appeared stronger for parental reports). However, and contrary to expectations, only self-reported affective empathy was negatively associated with aggressive behavior. Note that correlations involving social skills are Pearson but those referring aggression are Spearman, as this second variable did not follow a normal distribution.

**Table 7:** Zero-order correlations for empathy, social skills and aggression.

Measure	Social skills	Aggression	*N*
Cognitive empathy (BES-SR)	0.16*	-0.05	204
Affective empathy (BES-SR)	0.16*	-0.14*	204
Cognitive empathy (BES-PR)	0.32***	-0.06	237
Affective empathy (BES-PR)	0.25***	-0.01	237

*p < 0.05; ***p < 0.001.

## DISCUSSION

The aim of the current research was to study cognitive and affective empathy in middle childhood and early adolescence, and investigate their association with children’s family environment and social behaviors. For this purpose, we developed a European-Spanish adaptation of the BES, self-and parent-report forms. The psychometric properties of both measures of empathy were assessed using a representative population sample, including a wide range of socioeconomic backgrounds and a balanced gender distribution.

### THE STRUCTURE OF EMPATHY THROUGH THE SPANISH BES

Confirmatory Factor analyses run on the Spanish self- and parent-report BES forms brought out two empathy components that matched cognitive and affective empathy factors found respectively in the original BES version (Jolliffe and Farrington, 2006). Internal consistency indexes for the factors were considered satisfactory and were in the range of previous validations of the BES into other cultures (Albiero et al., 2009; D’Ambrosio et al., 2009; Geng et al., 2012). Further support for the two-componential structure of empathy through BES came from the significant but low (0.32 for self-report) to moderate (0.58 for parent-report) correlations between cognitive and affective empathy, which suggested that although related, each component explains a substantial non-shared portion of variance itself. Our findings show that the two componential model of empathy is valid in the European-Spanish culture, characterized as mainly collectivistic (Goodwin and Plaza, 2000; Gartstein et al., 2006), giving priority to the goals of the group, and emphasizing interpersonal contact and expression of feelings (Benet-Martinez and John, 1998). Taking into account the cultural variations provided by the countries in which the BES has been validated, altogether these results represent an important support for the generalizability of empathy as a construct across cultures and countries.

Minor adaptations were made in the process of validation of the Spanish BES with respect to the original instrument. Specifically, two items were excluded from the self-report form, and one more item from the parent form. Possibly, the low fit exhibited by some items could be explained by the young age of our sample. Specifically, the item 4 (“I get frightened when I watch characters in a good scary movie”) did not correlate with the Affective scale in the self-report form, and received a high frequency of “Not Applicable” option in the parent form, suggesting that children in the group of 6–8 years may not watch frightening movies. Interestingly, this item was also excluded in the Chinese validation of the BES (Geng et al., 2012), in which participants’ age ranged from 9 to 18 years. The other items excluded, item 6 (“S/he finds it hard to know when other children are frightened”) and item 7 (“I don’t become sad when I see other people crying”) are reversed ones; as pointed out by Juncos (1991), negative sentences are more difficult to understand than positive ones, so that children and parents could have found hard to understand and respond to these items.

### AGE AND GENDER DIFFERENCES IN CHILDHOOD AND EARLY ADOLESCENCE

Concerning age-related differences in the empathy components, some findings were consonant with theoretical expectations while others were not. On the one hand, affective empathy as reported by children and parents, showed stability throughout middle childhood and into adolescence. This is not surprising, considering that this component-referred to the emotional arousal that children experience in viewing others’ emotions-, can be considered a bottom–up process that depends on neural circuits whose development may asymptote early in childhood (Decety, 2010). In line with this interpretation, Dadds et al. (2008) found no age differences in the affective empathy when reported by parents of a sample of children aged 4–17 years, although the work by Geng et al. (2012) found age differences in the self-reported affective empathy in a sample of children aged 9–18 years old.

More controversial were the results concerning cognitive empathy, as older children reported higher scores than younger ones, whereas parents did not identify age-related differences. There are theoretical reasons to expect a higher capacity to comprehend others’ emotions throughout time, as the neural circuits implicated in emotion understanding partly overlap with those involved in Theory of Mind processing, especially the medial prefrontal cortex and right temporoparietal junction, and they still undergo maturation until late adolescence (Decety, 2010). In support to this expectation, other studies have found a tendency of cognitive empathy to increase from childhood and throughout adolescence (Dadds et al., 2008; Geng et al., 2012).

But why then did cognitive empathy scores reported by parents not increase with age? A possible explanation refers to the contexts or situations in which parents observe child’s behavior. Although BES cognitive and affective empathy cover children’s reactions to others’ emotions, including family members, friends, and unknown people, parents’ observations may be restricted to the familiar context, where even the youngest children may have shown high empathic responding. As suggested by Goubert et al. (2009), closer relationships (e.g., parent–child or other family relationships) are expected to elicit faster and stronger empathic responses than stranger or adversarial relationships. In support of this interpretation, parents in this study scored children’s empathy as higher compared to self-reported measures, and there is evidence that observers’ empathic reactions to other’s physical pain are stronger when suffered by a known person compared to one unknown (Bouchard et al., 2013). In concordance, Meyer et al. (2013) found different activation networks in processing other’ social suffering depending on the status of the observed person, with a friend’s suffering activating affective pain regions associated with the direct experience of pain, whereas observing a stranger’s suffering activated regions associated with thinking about mental states of others. It is important to note however, that although no significant fluctuations were found in cognitive empathy at the behavioral level, the subjective experience of empathy would surely be distinct at different ages, with more elaborated and complex processes as children mature into adolescence.

Concerning gender, differences between boys and girls have been found in self-reported affective empathy, with girls scoring higher than boys. This is very much in line with previous literature (Jolliffe and Farrington, 2006; Albiero et al., 2009; D’Ambrosio et al., 2009; Geng et al., 2012). As suggested by Han et al. (2008), these differences could be the result of how others’ emotions are processed, with males and females showing different patterns of brain networks activation when assessing their own emotional response to emotion expressed in other’s faces, as well as when they evaluate the emotional state expressed by other’s people face. Surprisingly, no gender differences were found in self-reported cognitive empathy. We think that this result could be explained in part by the early age of our sample; if as suggested by Eisenberg and Lennon (1983), differences in the empathic scales could reflect in fact internalized male/female stereotypes, it is possible that children in middle childhood and early adolescence be less susceptible to those socialization influences.

Contrary to our expectations, parent reports did not show any gender effects in either of the empathy scales. Arguments elicited in the previous paragraph alluding to an early age of our sample might also be applied to explain the general absence of gender differences here. Alternatively, following Levenson and Ruef (1992), it could also be suggested that since knowing and experiencing what another person is feeling are subjective qualities, parents in our sample could have had more difficulties identifying gender differences, as they have had to infer their children’s mental states in the light of their apparent behaviors. Nevertheless, our results do not fit with those of Dadd’s (Dadds et al., 2008), in which parents reported higher empathic skills for girls compared to boys. With these inconsistencies in mind, it is clear that the relationship between empathy and gender is complex, with different results depending on the age-period studied and the measurement instruments used. For that reason, more research incorporating different methodological approaches, including additional interviewing variables are needed to uncover the processes that interact with gender in the development of empathic behavior.

### CHILDREN’S EMPATHY IN CONNECTION TO FAMILY ENVIRONMENT AND SOCIAL BEHAVIORS

Cognitive and affective empathy positively correlated to family’s SES and emotional processes. This finding is supported by previous research where low SES was associated with lower levels of empathy (Jolliffe and Farrington, 2006). Similarly, higher scores in cognitive empathy were positively correlated to a more positive family environment, whereas affective empathy was negatively correlated to dissatisfaction with family relationships and with weaker parental management. This pattern of results was in line with several previous studies (e.g., Barnett, 1987; Garber et al., 1997; Lam et al., 2012) and highlights the relevance of family dynamics in empathy development. Families provide the essential context for children to learn the importance of interpersonal contact and concern for others, as well as the recognition, understanding, and sharing another’s emotional states.

This study also demonstrated that all empathy scales correlated positively with social skills. Higher cognitive and affective empathy were associated with more successful interactions with peers and adults in the contexts of home, school, and community. This result is not surprising given the reviewed literature in which children’s empathic skills were positively associated with their social adjustment (Eisenberg and Miller, 1987; Davis, 1996; Albiero et al., 2009; Eisenberg et al., 2010; Taylor et al., 2013). Interestingly, different results have been found depending on the informant when empathy and aggression were compared. Self-reported affective empathy was negatively associated with aggression. This was in line with the findings of Miller and Eisenberg (1988) in their review, in which affective empathy, measured by self-report, was negatively related to aggression. However, children’s empathy reported by parents was not associated with children’s aggression tendencies. This result is consistent with that reported by Geng et al. (2012), who found that the self-reported empathy scales were related to prosocial behavior, but not to behavior problems in a sample aged 9–18 years. Again, it is possible that the young age of the current sample is playing a role in these results. In support of this explanation, Lovett and Sheffield (2007) found strong relations between affective empathy and aggression in adolescence, but inconsistencies during the childhood period.

### PARENT–CHILD AGREEMENT IN EMPATHY PERCEPTIONS

Self- and parent-report forms of the Spanish BES developed in this work show a coherent view of empathy in middle childhood and early adolescence, with two components, cognitive and affective empathy, associated with relevant socio-emotional processes of children’s development, such as family climate and social behaviors. Nevertheless, the degree of convergence between children’s and parents’ measures was perhaps weaker than expected. As far as we are aware, this is the first study that examines the agreement between children’s and parents’ judgments using the BES, so that we do not have other research with which to contrast our results. This pattern of results could be due in part to the skewed distribution of parents’ report, as they tended to use the upper echelon of the scoring scale, probably affecting the degree of associations found. However, a low parent–child convergence is not overly surprising, and arguments concerning the nature of the phenomena and the context of observations could also explain it.

As previously mentioned, cognitive and affective empathy BES scales can be considered internal states, with parents and children perhaps using different strategies to respond to BES items, as the former had to infer their children’s mental states based on their children’s apparent behaviors, while the latter informed about their own reactions, leading consequently to a low parent–children convergence. In line with this interpretation, Cliffordson (2001) contrasted parent- and children-reported empathy scales measured through the IRI (Davis, 1980), finding significant correlations for the scales referred to affective outcomes (empathic concern and personal distress scales) while a non-significant correlation was found for perspective taking, considered as internal scale.

A second interpretation refers to the contexts or situations in which parents observe their children’s behavior. As mentioned above, parents’ observations may have been restricted to the context of the family, while children’s experiences in coping with others’ emotions could be much more varied. Differences in the contexts of observations have been used to explain the low level of agreement between therapists, parents, and children’s judgments in relation to behavioral problems diagnosis (Achenbach et al., 1987). In conclusion, parents’ and children’s reports can provide meaningful knowledge about children’ socio-emotional development, however, they cannot be considered equivalent but complementary sources of information. As suggested by Achenbach et al. (1987), no one informant can be replaced for another, but multiple methods and multiple informants are needed to address the validity and accuracy of psychological constructs.

In summary, the results found in this research lead us to consider the BES as a valuable instrument to measure both cognitive and affective empathy in a wide age range of Spanish-European children. Moreover, the parent-report form, developed in this work, allowed us to measure empathy in early middle childhood, when children still may not be able to reliably report on their empathic tendencies. This important development opens the way for the measurement of empathy in clinical/special populations through secondary sources. Nevertheless, a limitation of this study comes from the kind of measurement used, that is, empathy perceptions, which could be affected of possible biases, such as social desirability and gender stereotypes (D’Ambrosio et al., 2009), and future research should consider introducing a multi-method approach, including additionally structured observations altogether with electrophysiological techniques to contribute to a more comprehensive study of empathy.

## Conflict of Interest Statement

The authors declare that the research was conducted in the absence of any commercial or financial relationships that could be construed as a potential conflict of interest.

## Footnotes

1 There was no difference in the scale *Dissatisfaction With Siblings* by the number of siblings [*F*(6,101) = 1.83, *p* = 0.10].
